# Effects of Hydrosalpinx on Endometrial Implantation Failures: Evaluating Salpingectomy in Women Undergoing in vitro fertilization

**DOI:** 10.1055/s-0040-1722155

**Published:** 2021-02-18

**Authors:** Antonio Palagiano, Mauro Cozzolino, Filippo Maria Ubaldi, Chiara Palagiano, Maria Elisabetta Coccia

**Affiliations:** 1 Department of General and Specialized Surgery for Women and Children, Università degli Studi della Campania Luigi Vanvitelli, Napoli, Italy; 2 Department of Obstetrics, Gynecology and Reproductive Sciences, Yale School of Medicine, New Haven, Connecticut, United States; 3 Universidad Rey Juan Carlos, Madrid, Spain; 4 IVIRMA, IVI Foundation, Valencia, Spain; 5 GENERA Centre for Reproductive Medicine, Clinica Valle Giulia, Roma, Italy; 6 Università Campus Bio-Medico, Romq, Italy; 7 Department of Biomedical, Experimental and Clinical Sciences, Division of Obstetrics and Gynecology, Università degli Studi di Firenze, Azienda Ospedaliero Universitaria Careggi, Firenze, Italy

**Keywords:** hydrosalpinx, salpingectomy, endometrial receptivity, hydrosalpinx fluid

## Abstract

Hydrosalpinx is a disease characterized by the obstruction of the salpinx, with progressive accumulation in the shape of a fluid-filled sac at the distal part of the tuba uterina, and closed to the ovary. Women with hydrosalpinges have lower implantation and pregnancy rates due to a combination of mechanical and chemical factors thought to disrupt the endometrial environment. Evidence suggests that the presence of hydrosalpinx reduces the rate of pregnancy with assisted reproductive technology. The main aim of the present is review to make an overview of the possible effects of hydrosalpinx on in vitro fertilization (IVF). We conducted a literature search on the PubMed, Ovid MEDLINE, and Google Scholar data bases regarding hydrosalpinx and IVF outcomes. Hydrosalpinx probably has a direct toxic effect on sperm motility and on the embryos. In addition, the increasing liquid inside the salpinges could alter the mechanisms of endometrial receptivity. The window of endometrial receptivity is essential in the implantation of blastocysts, and it triggers multiple reactions arising from the endometrium as well as the blastocysts. Hydrosalpinx could influence the expression of *homeobox A10* ( *HOXA10* ) gene, which plays an essential role in directing embryonic development and implantation. Salpingectomy restores the endometrial expression of *HOXA10* ; therefore, it may be one mechanism by which tubal removal could result in improved implantation rates in IVF. In addition, salpingectomy does not affect the ovarian response, nor reduces the antral follicle count. Further studies are needed to establish the therapeutic value of fluid aspiration under ultrasonographic guidance, during or after oocyte retrieval, in terms of pregnancy rate and ongoing pregnancy.

## Introduction

Hydrosalpinx is the accumulation of fluid within the ampullar lumen as a result of occlusion of the infundibulum. It is a common condition among women of reproductive age, and it is related to diminished pregnancy rates (PRs) 1 2 3 with assisted reproductive technology (ART). The incidence of hydrosalpinx diagnosed by ultrasound among infertile women is between 10% and 13%, and it may increase to 30% with the use of hysterosalpingography or laparoscopy. 4

The diagnosis of hydrosalpinx is commonly made by ultrasonography (US) or hysterosalpingography, the latter representing the gold standard. However, under certain conditions, US is unable to detect small amounts of fluid inside the oviduct, which could consequently grow during ovarian stimulation, producing a deleterious effect on embryo implantation. 5 6 The exact mechanisms behind the adverse effects observed on pregnancy are not yet well understood. Hydrosalpinx fluid (HF) is known to be embryotoxic, and contains growth-factor inhibitors. Moreover, it seems that the liquid contained in the hydrosalpinx could reduce sperm motility and the velocity of motile spermatozoa following 24 hours of incubation. 7

The fallopian tube is distended during exogenous hormone administration, and this emphasizes that steroids affect the endoluminal ampullar secretion. 6 The aim of the present review was to evaluate the effects of hydrosalpinx on reproductive outcomes: it has a negative effect either on the embryo or the endometrium. In addition, we assessed the different surgical managements of hydrosalpinx.

## Methods

The present review included studies evaluating the effect of hydrosalpinx on IVF outcomes. The literature search was performed on the following databases: MEDLINE, EMBASE, Global Health, The Cochrane Library (Cochrane Database of Systematic Reviews, Cochrane Central Register of Controlled Trials, Cochrane Methodology Register), Health Technology Assessment Database, and Web of Science; we searched the entirety of those databses for studies published until July 2020. The literature search was conducted using the combination of the following Medical Subject Heading (MeSH) terms and any relevant keywords in different orders: “ *hydrosalpinx* ,” “ *hydrosalpinges* ,” “ *hydrosalpinx fluid* ,” “ *embryo toxicity* ,” “ *IVF* ,” “ *ART outcomes* ,” “ *implantation failure* ,” “embryotoxic,” “ *reproductive outcomes* ,” “ *live birth* ,” and “ *clinical pregnancy rate* .” The search was performed in English. The reference lists of the included studies were also manually checked to look for studies that were not found in the electronic literature search.

## Results

### Effects of Hydrosalpinx on Reproductive Outcome

The hydrosalpinges are associated with the presence of fluid in the uterine cavity, modifications in endometrial blood flow, the leukemia inhibitory factor, the inflammatory response, and *HOXA10* expression, 8 9 in the joint, which could explain the mechanism leading to decreasing PRs and increasing miscarriage rates in women undergoing ART. Many retrospective studies have shown an impaired outcome of IVF in the presence of hydrosalpinx, and the meta-analyses have demonstrated that the probability of achieving pregnancy in the presence of hydrosalpinx halved. 10 The miscarriage rate is increased in women with hydrosalpinx. 11 12 13 Theoretically, a good embryo could be implanted anywhere, as happens in the case if hysterectomized women, 14 however, perfect integrity of the endometrial cavity is required to maximize embryo implantation.

In recent years, the increased success rates of treatments using ART have as key factors embryo quality, endometrial receptivity, and embryo transfer as key factors. 15

The PR in most cases of IVF is below 40%; therefore, undiscovered pathologies in the uterus, endometrium and fallopian tubes could play a crucial role in implantation failure. Furthermore, some conditions, such as the presence of fluid within the uterine cavity, 16 17 could have a detrimental effect on endometrial receptivity. In particular, HF plays a role in reducing the PR in in-vitro fertilization programs. 11 Kassabji et al. 18 Demostrated the deleterious effects of HF on IVF outcome, and, a few years later, a meta-analysis by Camus et al. 3 demonstrated that the probability of achieving pregnancy in the presence of hydrosalpinx is halved, whereas the incidence of miscarriage is doubled. It is not well-known how the HF affects the implantation. It may be due to embryotoxicity, or to a direct effect on the endometrium, resulting in HF formation, or, finally, poor endometrial receptivity caused by alterations in the expression of markers or key molecules in the endometrium.

### Direct Embryo Toxicity

Beyler et al. 19 suggested a potential deleterious effect of the HF on in-utero embryo development. They evaluated in-vitro mouse embryo development in the presence of HF collected from 10 infertile women. The HF had a detrimental effect on the development of mouse embryos. Therefore, the poor PR in women with hydrosalpinx could be explained, in part, by the reflux of a lipophilic embryotoxic factor into the uterine cavity. 19 The HF may contain toxins that are potentially teratogenic. Morphological scores and the diameter of the yolk sac were significantly lower in embryos exposed to HF. 20

Roberts et al. 21 evaluated the effects of different concentrations of HF, with and without lactate supplementation, on the preimplantation development and implantation of murine models. The rates of development of balstocysts were respectively of 45%, 55%, 12.5% and 17.5% depending on the concentration of HF, and, with lactate supplementation, the rates were of 35.0%, 52.5%, 12.5%; without lactate supplementation, the rate was of 5.0%, while in the control group, it was of 63.8%.

The implantation rates for the 0.1% and 1.0% groups with lactate supplementation were of 43.0% and 25.0% respectively, and the rates for the groups with lactate supplementation were of 50.6% and 61.8%, while in the control group the implantation rate was of 65.5%. Hence, these results made it possible to hypothesize that the HF has a concentration-dependent decline in in-vitro murine embryo development, with a minimal effect on the implantation rates. Furthermore, lactate supplementation did not significantly influence the implantation rate at any HF concentration.

However, the embryotoxic effect of the HF has not been demonstrated in human embryos. 22 23 The analysis of the HF demonstrated the absence of bacteria, normal electrolyte concentrations with lower amounts of total protein and albumin, with a composition that does not differ from that of the normal tubal fluid. 22 All fertilized eggs showed physiological segmentation, and the embryonal development rate was not impaired by the HF. Even the granulosa cells incubated in HF showed the same steroidogenic capacity as those incubated in its absence.

In the same way, Strandell et al. 23 showed no negative effect on human embryonic growth: donated frozen embryos were incubated at concentrations of 50% and 100% of HF, but the HF had effects on blastocyst development.

In all likelihood, the low implantation rate in IVF patients with hydrosalpinx may not be due to an embryotoxic effect, and the differences regarding mouse embryos may be related to inter-species differences. Furthermore, the HF has a negative effect on sperm motility and survival after 24 hours of incubation, and the motility reduction depends on the concentration of HF. 7 Hydrosalpinx epithelial cells may be producing a fluid milieu which is hostile to sperm and early embryo development. 24

### Effects of Fluid Formation

Hydrosalpinx is associated with the presence of fluid within the endometrial cavity that may have a direct effect on the endometrium. The origin of the fluid is a point of controversy, but a leakage from the ampulla to the uterine cavity is the most accepted theory. Some authors have suggested that the reflux could have a “flushing” effect on the embryos, attributing to a mechanical interference with the implantation failure. 25 26 27 Before the invasion of the trophoblast, there is a progressive reabsorption of intraluminal uterine allowing the interactions between the embryo and the epithelial. 28 29 The HF has a direct, cytotoxic effect on developing mouse embryos, but not on human embryos, 19 21 22 23 in whom it reduces the implantation and pregnancy rates. The peaks of reabsorption of intraluminal uterine fluid occur at the expected time of implantation in rodents, 30 31 and are controlled the by ovarian hormone secretions, local ion and water channels. The mechanisms underlying the fluid formation in the hydrosalpinx are poorly-known or not consistent. 32

The involvement of epithelial transporters and ion channels of the fallopian tube is a possibility, particularly the cystic fibrosis transmembrane conductance regulator (CFTR). In pelvic inflammatory disease (PID), which is mainly caused by infection by *Chlamydia trachomatis* , CFTR-mediated events may culminate in hydrosalpinx formation, 33 34 and there is evidence 35 available that shows that ∼ 20% of woman affected by chlamydial lower-genital-tract infection develop PID. A shift between the chlamydial and endometrial infections was reported in a study by Jones et al., 36 in which *C. trachomatis* was recovered from uterine and fallopian tubes in women affected by acute salpingitis. 37 In both cases, there was a higher incidence of miscarriage rates 38 and higher levels of chlamydial heat shock proteins (HSPs), which seem to be responsible for the local immune response followed by an inflammatory reaction and poor embryo implantation. 39 Spandorfer et al. 40 demonstrated that HSP antibodies were more prevalent in women with hydrosalpinx and tubal occlusion than in patients with male factor infertility.

Furthermore, Witkin et al. 39 reported that the immunoglobulin A (IgA) antibodies against Chlamydia detected in the uterine cervix dramatically correlated to lower PRs in women undergoing IVF and embryo transfer (IVF-ET).

**Fig. 1:**
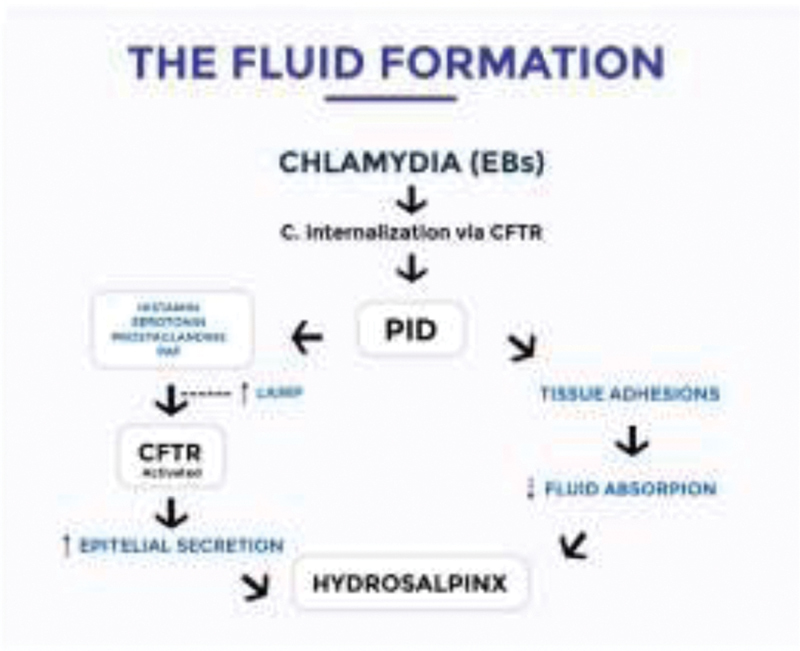
Mechanisms responsible for the formation of the hydrosalpinx fluid.

Although the connection between PID, Chlamydia infection and hydrosalpinx is largely recognized, the sequence of events that leads to the formation of fluid is still debated and not clearly demonstrated ( Fig. 1 ).

It is known that the movement of water in the oviducts, which does not depend on active transport, may represent the response to osmotic gradients due to the transportation of ions. 41 In particular, chloride ions move actively in the direction of the oviduct lumen, from the tubal serosa to the tubal mucosa, 42 43 and generate a transepithelial potential difference across cultured cells. 44 45

The inner mucosa of the fallopian tubes is lined with ciliated columnar epithelial cells and peg cells (non-ciliated secretory cells). The loss of membrane polarity could generate HF after PID. Sodium/hydrogen exchangers (NHEs) and anion Cl ^−^ /HCO3 ^−^ (AE), well-known plasma membrane transporters in human epithelial cells, are involved in this process. The CFTR has a well-recognized function as a cyclic adenosine 3′,5′-monophosphate (cAMP)-activated chloride channel. The increases in the levels of cAMP are followed by an increase in the transepithelial potential difference in current, leading to enhancements in fluid secretion. 46 The CFTR is a transporter of chloride ions; furthermore, it acts as a receptor for some bacteria in the epithelial cells. *Pseudomonas aeruginosa* binds to the CFTR in pulmonary epithelial cells, whereas *Salmonella typhi* and *Cholera vibro* bind to the CFTR in gastrointestinal cells, increasing the serosa-to-mucosa fluxes of sodium and chloride, with subsequent diarrhea. 47 48

**Fig. 2:**
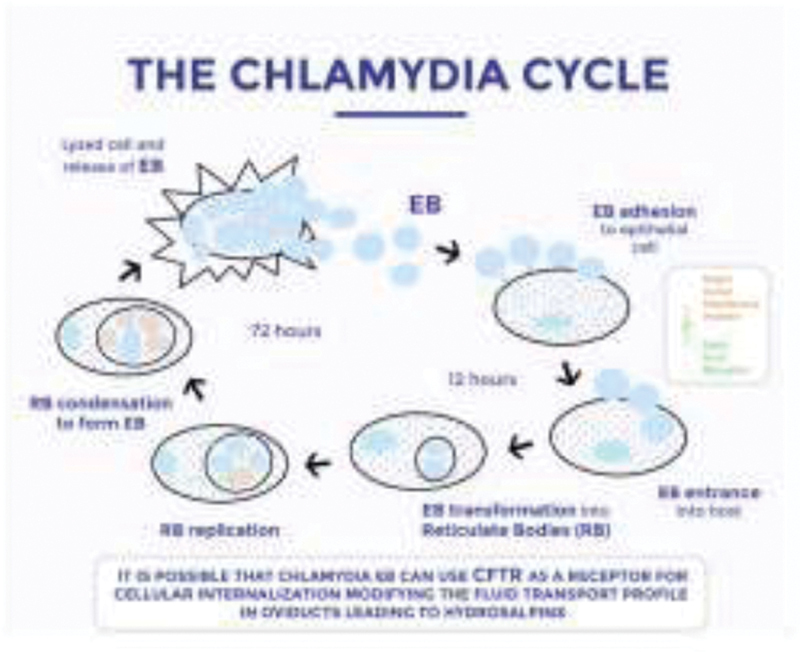
Mechanisms through which the chlamydia cycle occurs within infected cells.

It is possible that Chlamydia elementary bodies (EBs) can use the CFTR as a receptor for cellular internalization in female infections of the reproductive , modifying the profile of the fluid transport in the oviducts, leading to hydrosalpinx ( Fig. 2 ). Downing et al. 49 studied the effects of inflammatory mediators, which may increase microvascular permeability, leading to fluid formation by the tubal epithelium. Histamine, serotonin, prostaglandins and the platelet-activating factor (PAF) are involved in this process. In particular, histamine, in cultured epithelial cells, influences ion movements and tubal secretions of fluid. In the presence of fimbrial adhesions, which are the consequence of infection and inflammatory disease, the epithelial secretion is blocked within the oviduct, leading to the formation of hydrosalpinx. 49

The tumor necrosis factor (TNF) may be involved in the pathogenesis of acute tubal injury associated with Chlamydia infection. In fact, in women who are culture-positive for *C. trachomatis* , the TNF was identified only in those fallopian tubes with visual evidence of the disease, while the fluids obtained from morphologically normal tubes were negative. Therefore, localized cell-mediated activation of the immune system, which is identified by TNF production, appears to be a typical component of salpingitis. 38

Endometrial integrins may also modify endometrial receptivity. Meyer et al. 50 studied 103 patients affected by hydrosalpinx who underwent endometrial biopsies during the window of implantation, searching for three integrins as markers of endometrial receptivity: α _1_ β _1_ , α _4_ β _1_ , and α _v_ β _3_ . Women with hydrosalpinx expressed significantly lower levels of α _v_ β _3_ compared with the control group, while there was no difference in the expression of the other two integrins. From a histological point of view, the endometrium with an absence of expression of α _v_ β _3_ was significantly out of phase compared with the control group. Furthermore, women biopsied after hydrosalpinx removal demonstrated an increase in α _v_ β _3_ expression in 70% of the cases. The present study confirms that a lower implantation rate in hydrosalpinx may be caused by poor endometrial receptivity, and that the tubal surgical removal in these patients can improve the IVF-ET outcome.

### Poor Endometrial Receptivity

The window of endometrial receptivity is essential in the implantation of blastocysts, and it triggers multiple reactions from the endometrium as well as the blastocysts. 51 The receptive endometrium and stimulated blastocysts function simultaneously to achieve blastocyst implantation, leading to pregnancy. 52 Poor endometrial receptivity may be due to an abnormal expression of key molecules in the endometrium, cytokines, steroid hormones, peptides, growth factors and enzymes, which are essential for implantation. 53 The dialogue between the endometrium and the embryo is mediated by the expression of certain cytokines and other substances during the implantation window. The *HOXA10* is a protein-coding gene that plays an essential role in directing embryonic development and implantation. 54 55 Its expression is necessary for endometrial receptivity. 55 56 57

A targeted disruption or a target mutation of the *HOXA10* gene in mice causes implantation failure, but the same mice produce good embryos that normally implant in a wild-type surrogate. In contrast, wild-type embryos fail to implant in *HOXA10* deficient mice. 58 The *HOXA10* is expressed in the uterus during the menstrual cycle, and its expression increases during the mid-secretory phase of the menstrual cycle, corresponding to the time of implantation. In this “window,” the levels of expression of the *HOXA10* increase dramatically in the glands, and even in the stromal cells. 59 60 61 62 In cultured endometrial cells, the expression of HOXA10 was stimulated by estrogen or progesterone with a concentration-dependent dose, for progesterone. 56 Reduction of maternal HOXA10 results in proportionally diminished windows of implantation. Furthermore, altered levels of HOXA10 expression regulate the degree of endometrial receptivity. 63

The *HOXA10* gene influences embryo implantation through several mechanisms, such as pinopod development, 64 leukocyte infiltration, and stromal decidualization. 62 65

A defect in the endometrial expression of the *HOXA10* gene is present in several aberrations and pathologies involving the uterus, ovaries and salpinges, such as endometriosis, submucosal uterine leiomyomas, polycystic ovary syndrome (PCOS) and hydrosalpinges. In fact, optimal anatomy and physiology are necessary for normal implantation and placentation. 8

The expression of the *HOXA10* was significantly lower in infertile patients with hydrosalpinges compared with controls with fertile women. Salpingectomy resulted in a statistically significant 15-fold increase in endometrial *HOXA10* expression. 66

The present research demonstrates that the decrease in *HOXA10* expression in response to HF may constitute a potential molecular mechanism for diminished implantation rates. Salpingectomy restores endometrial *HOXA10* expression, resulting in improved implantation rates in IVF-ET.

### Management of Hydrosalpinx

Hydrosalpinx has a negative effect on the outcome of IVF; therefore, any surgical intervention performed on the damaged tube should increase the chances of a successful outcome. 67

Johnson et al. 68 published a complete review in 2010 that compared the surgical treatments on the fallopian tubes, prior to IVF, in women with hydrosalpinx. The authors included five randomized controlled studies involving 646 women, regarding salpingectomy, laparoscopic tubal occlusion, ultrasonographic aspiration of HF before or during the oocyte collection and, finally, salpingostomy. The results showed that the odds of ongoing pregnancy and clinical pregnancy increased after laparoscopic salpingectomy, prior to IVF, for patients with hydrosalpinx. 68 Tubal occlusion compared with salpingectomy did not show a significant advantage in terms of ongoing pregnancy or clinical pregnancy.

The Practice Committee of the American Society for Reproductive Medicine (ASRM), in collaboration with the Society of Reproductive Surgeons (SRS), published a study about IVF outcomes in women with unilateral or bilateral hydrosalpinges. 69 The PR in patients with hydrosalpinges was lower than that of the control group, with identical negative effects in both fresh and frozen embryo transfer cycles. Even the miscarriage rate was 2.3 times higher among women with hydrosalpinx than among those unaffected. Preliminary laparoscopic salpingectomy or proximal tubal occlusion, prior to IVF, improves pregnancy and live birth rates, whereas data was insufficient to evaluate the effectiveness of alternative treatments such as laparoscopic neosalpingostomy, transvaginal aspiration of HF, hysteroscopic tubal occlusion, or antibiotic treatment. Transvaginal aspiration of HF during egg collection is still controversial. 70 71 The data in the literature is insufficient to determine whether hysteroscopic tubal occlusion could be considered helpful in preventing the deleterious effect of HSF. A recent study reports that the placement of Essure (Bayer AG, Leverkusen, Germany) is an alternative method for fallopian tube occlusion in hydrosalpinx before IVF when laparoscopy is contraindicated. 72

The hysteroscopic proximal occlusion by intratubal devices improves the chance of achieving clinical pregnancy compared with no intervention. Nevertheless, the Essure implant is associated with a higher miscarriage rate compared with the other interventions. 73 Unfortunately, insertion of the Essure in the management of hydrosalpinx prior to IVF remains off-label, although the implant could increase fluid within the uterine cavity, with a consequent lower PR. 74 In fact, the hysteroscopic placement of Essure devices before IVF produces inferior pregnancy rates compared with the laparoscopic approach. 75 Therefore, salpingectomy should be the first option for women with hydrosalpinx before undergoing an IVF cycle. Salpingostomy should be considered to conserve the fallopian tubes, enabling natural conception. 76 77

## Conclusion

Hydrosalpinx is associated with poor IVF outcomes, with reduced live birth rates and higher pregnancy loss. Therefore, accurate diagnosis of hydrosalpinx are critical, especially when a tiny amount of fluid inside the Fallopian tubes may be almost undetectable. The surgical removal of hydrosalpinx prior to IVF improves PRs and reproductive outcomes. 78 In addition, salpingectomy does not affect the ovarian response, nor reduces the antral follicle count. 79 Laparoscopic salpingectomy is the optimal surgical approach; therefore, it should be recommended. Further studies are needed to establish the therapeutic value of HF aspiration under ultrasonographic guidance, during or after oocyte retrieval, in terms of PR and ongoing pregnancy.
